# Plant Immunity: At the Crossroads of Pathogen Perception and Defense Response

**DOI:** 10.3390/plants13111434

**Published:** 2024-05-22

**Authors:** Sajad Ali, Anshika Tyagi, Zahoor Ahmad Mir

**Affiliations:** 1 Department of Biotechnology, Yeungnam University, Gyeongsan 38541, Republic of Korea; anshikabio@yu.ac.kr; 2 Department of Plant Science and Agriculture, University of Manitoba, Winnipeg, MB R2M 0TB, Canada; zahoorbio@gmail.com

**Keywords:** plant immunity, pathogens, receptors, signaling, calcium, reactive oxygen species, hormonal crosstalk, disease resistance

## Abstract

Plants are challenged by different microbial pathogens that affect their growth and productivity. However, to defend pathogen attack, plants use diverse immune responses, such as pattern-triggered immunity (PTI), effector-triggered immunity (ETI), RNA silencing and autophagy, which are intricate and regulated by diverse signaling cascades. Pattern-recognition receptors (PRRs) and nucleotide-binding leucine-rich repeat (NLR) receptors are the hallmarks of plant innate immunity because they can detect pathogen or related immunogenic signals and trigger series of immune signaling cascades at different cellular compartments. In plants, most commonly, PRRs are receptor-like kinases (RLKs) and receptor-like proteins (RLPs) that function as a first layer of inducible defense. In this review, we provide an update on how plants sense pathogens, microbe-associated molecular patterns (PAMPs or MAMPs), and effectors as a danger signals and activate different immune responses like PTI and ETI. Further, we discuss the role RNA silencing, autophagy, and systemic acquired resistance as a versatile host defense response against pathogens. We also discuss early biochemical signaling events such as calcium (Ca^2+^), reactive oxygen species (ROS), and hormones that trigger the activation of different plant immune responses. This review also highlights the impact of climate-driven environmental factors on host–pathogen interactions.

## 1. Introduction

Plants face different microbial pathogens, such as fungi, bacteria, oomycetes, and viruses, which affect their growth and reproduction [1]. Microbial diseases are one of the leading causes of crop yield losses in modern agriculture and have significant global repercussions on food security, economy, and environmental sustainability [2,3]. For example, they can reduce yield production by up to 16%, which is further increased during post harvesting [4]. Pathogens can spread to plants by different modes such as water, air, and transmission by insects, animals, and humans. They utilize diverse strategies to infect plants, including immune suppression and the secretion of toxins and degradative enzymes that aid in colonization and nutrient release [1,5]. Some pathogens may directly enter and infiltrate plant tissues, whereas others enter through wounds or natural openings. Fungal pathogens have different modes of nutritional lifestyles, such as biotrophic, hemi biotrophic, and necrotrophic, and they evolve different strategies to infect plants [3]. Necrotrophic fungal pathogens obtain their energy from dead or dying cells, whereas biotrophs obtain their nutrients and energy from living cells. In contrast, hemibiotrophs first infiltrate living cells before switching to a necrotrophic way of life in order to harvest nutrients from the dead tissues [3]. Oomycetes and fungal pathogens use special structures like appressoria and haustoria to penetrate host cells and to release effectors as well as to obtain nutrients [5]. For instance, the smut disease-causing fungus *Ustilago maydis* secretes the Pep1 effector from fungal hyphae, which is necessary for host tissue penetration [6]. On the other hand, viruses are obligatory parasites that require a host cell to proliferate and infect plants. During plant virus interaction, viral pathogens hijack the host machinery system, leading to metabolic, physiological, molecular, and morphological alterations in plants [7]. Particularly, viral proteins play a major role in pathogenesis in addition to replication, encapsidation, and transmission [8]. Bacterial pathogens use different strategies to infect plants. For instance, they use different secretion systems to secrete effectors both within and outside of plant host cells. The type III secretion system (T3SS), which transports effectors within host cells and is essential for pathogenesis, is a well-studied secretory pathway for bacterial effectors [9]. For example, HopM1, an effector from *Pseudomonas syringae,* targets the *Arabidopsis* 14-3-3 protein GRF8/AtMIN10, suppressing stomatal defense [10]. Through their stylet secretions, insect pathogens like psyllids and aphids can also transfer effectors during feeding. Some of the typical signs of plant disease in plants are necrosis, wilting, rot, deformation, mold, discoloration, pustules, hypertrophy and hyperplasia (overgrowth), mummification, and destruction of infected tissue [11].

Primary pathogens in plants can also trigger host susceptibility to secondary infections by suppressing their immune system, which can further deteriorate their growth and survival. For instance, when the foliar bacteria *P. syringae* infects Arabidopsis, the plants become highly susceptible to the necrotrophic fungal pathogen *Alternaria brassicicola* [12]. Similarly, for biotrophic pathogen *Albugo candida*, infection in *Arabidopsis thaliana* suppresses the immune system, making them more susceptible to avirulent pathogens [13]. In some cases, various pathogen-produced molecules have been identified that suppress the plant immune system during co-infection. For example, in Arabidopsis, the natriuretic peptide receptor NPA produced by *P. syringae* downregulates a wide range of defense-related genes, enabling subsequent infection by the virulent *A. brassicicola* [13,14]. In a similar vein, fusaric acid released by *F. oxysporum* inhibits the expression of genes that control 2,4-diacetylphloroglucinol’s antimicrobial action and makes wheat more susceptible to *Pseudomonas fluorescens* infection [15]. Pathogens can also alter the physiology, metabolism, and resource availability of their host plant, which can have a direct impact on plant development and fitness. As part of their virulence approach, they can control plant growth by manipulating plant hormone signaling or by mimicking phytohormones. For instance, bacterial pathogens can alter root growth by regulating auxin signaling [16]. Fascinatingly, lateral root development was greatly stimulated by *P. syringae* pv. tomato DC3000 infection. The development of lateral roots produced by *P. syringae* pv. tomato requires the presence of ARF19 and auxin response factor 7 (ARF7). However, salicylic acid (SA) inhibits lateral root formation and blocks the entry of *P. syringae* pv. tomato. On the other hand, a variety of developmental abnormalities, such as a thin lamina, a serrated leaf border, and an uneven leaf surface, were seen in *Arabidopsis* infected with the bacterial pathogen *Rhodococcus fascians* [17]. These developmental changes by *R. fascians* were due to the modulation of the host cytokinin (CK) metabolism, triggering cytokinin (CK) production through Arabidopsis response regulators 5/cytokinin 5 (ARR5/CK5) signaling [18]. For successful infection, pathogens can also utilize host nutritional resources that are required for normal plant growth and development [19]. Additionally, they produce diverse virulence factors that affect the plant primary metabolism, namely photosynthesis, which leads to growth retardation [20].

## 2. Impact of Climate Change or Environmental Factors on Plant–Pathogen Interaction

**Figure 1:**
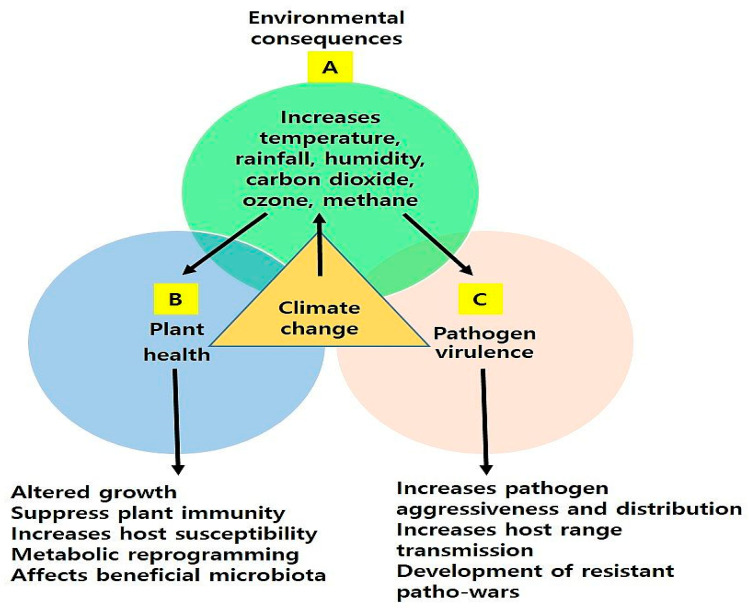
A schematic illustration showing the effect of climate change on environmental factors (A), pathogens (B), and the host defense system (C). Climate change increases temperature, rainfall, humidity, drought, carbon dioxide, and methane, which affects the plant health and immune system. These factors also change pathogen distribution, virulence, and resistance.

Plant pathogens are diverse in nature, and their interactions with their respective hosts are influenced by environmental factors [21]. In plant pathology, the well-known “disease triangle” concept emphasizes how pathogens and plants interact with their environment. Three main factors—pathogen virulence, host vulnerability, and ideal environmental conditions—determine the development of disease in plants [22]. Any alterations in favorable environmental conditions can affect disease development in plants. Environmental factors like temperature, water availability, light, carbon dioxide, and nutrients in the soil directly affect plant–pathogen interactions, disease susceptibility, and pathogen distribution [23,24]. For example, drought stress affects plant–pathogen interactions and disease development in plants. Rice subjected to mild drought circumstances has increased *Magnaporthe grisea* susceptibility, which is due to the downregulation of plant defense marker genes such as pathogenesis-related genes [25]. In wheat, drought stress enhanced disease development caused by *Fusarium* spp. [26]. Plant fungal pathogens thrive at temperatures between 15 to 24 °C, and variations in the average global temperature will result in the establishment of increasingly pathogenic strains. According to Shakya et al. [27], variations in temperature have an impact on the development of the *Phytophthora infestans* that cause potato late blight disease. In wheat, the rising temperatures have led to the development of more virulent *Puccinia striiformis* race globally, which can have a more detrimental effect on crop productivity [28]. Similarly, in chestnut, increased winter temperatures have enhanced disease development and increased mortality [29]. On the other hand, higher levels of carbon dioxide have increased the *Fusarium graminearum*, virulence, and disease development in susceptible and resistant wheat cultivars [30]. Many studies have predicted that climate change will change temperature, water availability, and CO_2_ concentration, which can have a dramatic impact on pathogen distribution, virulence, and host defense responses [23,27]. The recent events in climate change have evolved novel pathovars. For instance, the climate-driven shift towards heavier rainfall, elevated mean winter temperatures, and precipitation transition from summer to winter all contribute to an increased susceptibility to Phytophthora species [31]. It is anticipated that the global temperature increase will have a positive impact on pathogen evolution and disease distribution. One of the main abiotic drivers of climate change is temperature elevation, and models have indicated that this will lead to an increase in the frequency and intensity of disease epidemics [21]. Climate change, especially warming nights and reduced frost weather conditions, has led to the increase in pathogen virulence and disease occurrence [32]. Plant fungal pathogens thrive at temperatures between 15 to 24 °C, and variations in the average global temperature will result in the establishment of increasingly pathogenic strains. Similarly, a 10-degree temperature variation is ideal for soybean rust infection to cause maximum damage. As the climate shifts, new strains that are more adapted to survive will appear and take dominance. For instance, recent studies on the potato disease *P. infestans* and the wheat pathogen *Zymoseptoria tritici* have shown that both pathogens are well adapted to climatic fluctuations [33,34]. This adaptation is related to modifications in both genomic structure and gene expression. Further, we show the impact of climate change on plant–pathogen interactions in Figure 1. The recent development in statistical data-analyzing tools based on artificial intelligence prediction models have helped researchers to understand disease infestation and host specificity. However, future studies are required to develop new models to study how climate-change-driven factors can influence pathogen distribution, aggressiveness, and virulence and host specificity. Also, how they will affect host immune responses should be the top priority to tackle among researchers to combat future disease outbreaks.

## 3. Pathogen Perception and Plant Immunity

The ability of plants to perceive and respond to pathogens governs the outcome of plant–pathogen interactions. It is well documented that plants have evolved many defense mechanisms to restrict pathogen invasion. The initial line of plant defense against pathogen attack is made up of preformed elements found on the surface of plant organs, such as the wax layer, cuticular lipids, hard cell walls, antimicrobial enzymes, or secondary metabolites [35,36]. Pathogens can overcome the preexisting defensive layer and are confronted by plants’ inducible defense responses [37,38]. Generally, plants’ extensive repertoire of immunological receptors that are able to identify any type of pathogen and their derived elicitors triggers the plant’s inducible defense responses [38]. For successful infection, pathogens must overcome physical barriers, evade or suppress immune perception, and derive nutrients from plant tissues [38,39]. However, the plant immune system uses different strategies to defend from pathogen attack [40]. The first reaction is the pathogen or their derived molecule or effectors recognition by immune receptors like extracellular pattern-recognition receptors (PRRs) and nucleotide-binding leucine-rich repeat (NLR) receptors, which leads to the activation of diverse defense signaling pathways PTI and ETI to defend from the pathogen attack. The identification of R genes from plants and Avr genes from pathogens marked the beginning of the development of the molecular model of plant immunity [41,42,43,44,45]. Later, in 2000, the first plant receptor for a pathogen elicitor was discovered. Based on these findings, two tiers of plant immunity were proposed, namely pattern-triggered and effector-triggered immunity (PTI and ETI) [37]. Flagellin Sensing 2 (FLS2) was the first PAMP cell surface receptor identified in Arabidopsis that can recognize flg22 [46]. PRRs include receptor-like kinases or receptor-like proteins, which have different extracellular ligand-binding domains, including malectin-like domains, lectin domains, leucine-rich repeat (LRR) domains, and LysM domains, which function as mediators of the pathogen or pathogen-derived PAMPs and DAMPs recognition [47]. For example, pathogen protein and peptide patterns or phytocytokines generated from plants are sensed by LRR ectodomain (ECD) receptors; pathogen oligosaccharides or carbohydrate structures are recognized by lysin-motif ECD receptors; and microbial lipids are preferentially bound by lectin ECD receptors. Both RlKs and RlPs have a single helical transmembrane domain, RKs feature an intracellular protein kinase domain for signaling, and RPs have a short cytoplasmic tail [47]. In addition to pathogen recognition, RLKs and RLPs also play important role in plant abiotic and mechanical stress perception as well as growth regulation. The two most common kinds of plant PRRs are cell surface leucine-rich repeat domain (LRR) receptor kinases (LRR-RKs) and LRR receptor proteins (LRR-RPs). Activation of RLKs leads to a series of biochemical changes, such as mitogen-activated protein kinase (MAPK) phosphorylation, which further triggers calcium burst, ROS wave formation, callose deposition, activation of hormonal signaling pathways, and transcriptional reprogramming of plant defense genes [48]. We display different PRRs identified in plants that act as key receptors for pathogen or MAPs/DAMPs recognition in Table 1.

**Figure 2:**
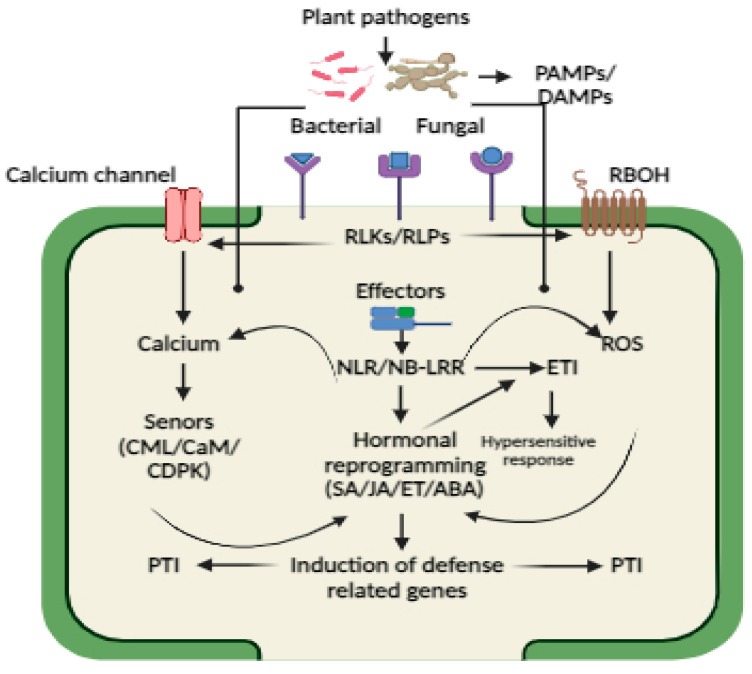
Schematic illustration showing the activation of two-tier plant immunity, namely PTI and ETI, in plants after pathogen, MAMPs/DAMPs, or effectors perception by PPRs and NLRs. Plants undergo biochemical reprogramming such as calcium burst, ROS production, and hormonal activation, which regulates diverse antimicrobial responses like hypersensitive response or programmed cell death or systemic acquired resistance.

On the other hand, intracellular NLRs can recognize diverse effector proteins that are incorporated into plant cells during pathogen invasion, resulting in the activation of ETI. In plants, three types of NLRs, namely Toll-interleukin-1 receptor homology (TIR) domain containing NLRs (TNLs) and coiled-coil (CC) domain containing NLRs (CNLs) and resistance to powdery mildew 8 (RPW8)-like CC domain (CC-R)-containing NLR (RNL), have been identified that can sense pathogen effectors [91]. Different NLR subtypes oligomerize into resistosome structures upon activation, fulfilling dual functions in signal transduction and pathogen identification. ETI is associated with localized programmed cell death, also called hypersensitive response (HR-PCD). SA and ROS are two important signaling components that have been shown to activate ETI triggered PCD, which can inhibit the spread of pathogens to neighboring cells [92]. However, PCD is regulated by SA-dependent non-expresser of pathogenesis-related protein 1 (NPR1) via the activation of plant defense genes and the forming of SA-induced NPR1 condensates (SINCs) in the cytoplasm, which sequester and degrade various signaling components involved in cell death, thereby turning on the pro-survival immune response [92]. How PPRs and NLRs triggers biochemical reprograming after pathogen or effector recognition, leading to the activation of inducible plant defense, is shown in Figure 2.

In plants, both PTI and ETI elicit a systemic defensive response known as systemic acquired resistance (SAR), which provides a broad spectrum of disease resistance for a longer time [93]. SA accumulation is essential for the activation of SAR pathway in plants, and SA degradation by the bacterial SA hydroxylase *NahG* results in failure of SA-mediated resistance and SAR formation [94]. Despite the fact that SAR may be induced exogenously without the need for an ETI by applying SA and its synthetic analogs, how ETI triggers systemic SA accumulation is not fully understood. Recently, it was found that RBOHD produced H_2_O_2_, acting as a mobile signal for the formation of systemic SA by modulating the activity of its biosynthesis genes like *ICS1* via the sulfenylation of the CCA1 HIKING EXPEDITION (CHE) transcription factor (TF). It is noteworthy that plants with mutations in their H_2_O_2_-sensitive cysteine residue in CHE no longer produce SAR or accumulate SA systemically [95]. SAR in plants can persist for several weeks to months and can provide a broad spectrum of disease resistance without causing cell death. This is associated with massive transcriptional reprogramming and is dependent on *NPR1* and other transcription factors like TGAs. The accumulation of PR proteins is the hallmark of SAR, which possess diverse antimicrobial activity.

Autophagy has emerged as an important component of plant immune response, which regulates hormonal levels and hypersensitive response. In general, autophagy is catabolic process that transports damaged organelles or undesired proteins to vacuoles where they are broken down and recycled [96]. It is crucial for the control of plants’ cellular homeostasis, cell death, and stress adaption [96]. So far, 40 autophagy-related (ATG) genes have been found in plants, and they all have different but complementary functions in promoting autophagy [97]. In plant immunity, autophagy can have a dual function, supporting both pro-cell-death and pro-cell-survival processes [98]. For instance, autophagy can play key role in inhibiting the spread of PCD to surrounding cells during the ETI response [98]. Previous research has shown that the silencing of the autophagy-associated gene *ATG6/Beclin1* in tobacco plants results in a substantial spread of HR-PCD into nearby healthy tissue and systemic leaves during tobacco mosaic virus (TMV) infection. This study also reported that silencing other autophagy associated genes like *ATG3, ATG7*, and *VPS34* also showed the same results, which further supports that autophagy protects uninfected or healthy plant cells during HR response [98]. Autophagy can also protect uninfected plants from necrotrophic cell death. For instance, *Arabidopsis* ATG6 RNAi lines showed unconstrained spread of disease-induced cell death after infection with pathogenic Pst DC3000 [99]. Similarly, the silencing of autophagy genes such as *atg5-1*, *atg10-1*, and *atg18a-1* in Arabidopsis triggers disease-induced cell death during *A. brassicicola* infection [100]. These studies provide evidence on the involvement of autophagy in plant immunity; however, there remain many knowledge gaps on understanding the molecular underpinning of its regulatory mechanism during different plant–pathogen interactions. Therefore, future studies are required to identify potential molecular players that control autophagy during PCD and disease-induced cell death.

RNA silencing or RNA interference (RNAi) is also an important plant defense response that protects plants from pathogen infection [101]. It was initially shown that RNA silencing in plants occurs as a post-transcriptional process during viral infection and transgenesis [101]. There are two types: RNA transcriptional gene silencing (TGS) and post-transcriptional gene silencing (PTGS), and double-stranded (ds) or hairpin RNA substrates of dicer (DCL in plants) are important intermediary molecules that initiate RNA silencing to direct RNA degradation, DNA methylation, and translational repression [102]. Plant immunity is precisely regulated by small noncoding RNAs (sRNAs), which are important modulators of gene expression. The two main groups of plant sRNAs are small interfering RNAs (siRNA), which are recognized for their functions in silencing viral RNAs, and microRNAs, which modulate diverse immune and growth responses [103]. But unlike bacterial and fungal infections, viral genomes proliferate inside of their hosts, which is why RNA-silencing pathways are essential for anti-viral defense. Plants that are infected with any type of virus or subviral agent, such as viroids, satellites, or faulty RNAs, produce more viral siRNAs that may then be used to drive silencing against the viral genome [104]. Consequently, viruses are both targets and inducers of RNA silencing. Recent studies have shown that siRNA can also repress bacterial, fungal, and oomycete infection by targeting pathogen genes [105]. The identification of RNA-silencing suppressors in plant pathogens implies that host-silencing disruption is a common virulence tactic used by numerous phytopathogens [103]. Although there are many reports on the role of RNA silencing in combating pathogens, there remain many knowledge gaps on how pathogens suppress RNA silencing, therefore necessitating future investigation. In the future, it will be interesting to explore the how pathogens suppress RNA-silencing defense response in plants to promote disease and their multiplication. Also, identification of anti-RNA-silencing virulence factors in bacterial fungal and oomycetes pathogens can pave the way for improving disease resistance in plants.

## 4. Role of Calcium and ROS in Plant Immunity

After pathogen or effector recognition by different exterior and interior receptors, cells undergo biochemical reprograming like calcium burst, ROS wave formation, and defense hormonal activation, which modulate different immune responses (Figure 2). Both ETI and PTI activation triggers a variety of signaling events that are mostly similar, such as Ca^2+^ fluxes, ROS burst, transcriptional reprograming, and phytohormone production, with ETI exhibiting a stronger response than PTI [106]. The early signaling events are an accumulation of secondary messengers like calcium and ROS that act as biochemical language codes that are sensed by different sensors that decode and elicit a series of downstream signaling cascades [106]. Previous studies have shown a mutual interplay between calcium and ROS, which has a positive influence on plant defense signaling [107,108].

Calcium signaling is reported to be essential for both layers of the plant immune system since alterations in intracellular Ca^2+^ levels have been well documented following both PRR and NLR activation [107,108]. However, plant cells need to maintain low cytosolic Ca^2+^ levels due to its cytotoxicity. Therefore, Ca^2+^ is sequestered in intracellular stores, such as the apoplast or the vacuole and endoplasmic reticulum in plants, but it can also be stored in vesicular compartments, mitochondria, and chloroplasts through active transport, which creates massive electrochemical potential gradients across membranes [109,110,111]. Ca^2+^ signals are produced by the coordinated activity of active transporters and channels, and they entail intracellular store release and apoplast inflow. Interestingly, various calcium channels, such as cyclic nucleotide-gated channels (CNGCs) [112], glutamate receptor-like (GLRs) [113], and hyperosmolality-induced channels (OSCAs) [114], have been identified to play a key role in PTI-mediated calcium-dependent signaling. In contrast, Ca^2+^ channels found in ETI require the formation of multimeric NLR resistosomes that form pore structures in the plasma membrane from the cytosolic side. We detail the roles of different calcium channels in plant immunity in Table 2.

It is evident that Ca^2+^ influx across the plasma membrane is essential in both levels of immunity since Ca^2+^ channel blockers that stop Ca^2+^ entrance from the apoplast reduce Ca^2+^ signals and immunological responses in both PTI and ETI [97,98]. Also, gene-knockout studies have revealed that blockage of calcium channels directly affects the plant defense response’s against pathogens [113,121]. However, there remain many knowledge gaps on how pathogens trigger calcium channel activation and the role of precise calcium sensors during immunity activation [123]. Future research is required to determine how RLks and RLPs contribute to the activation of calcium channels during pathogen attack. It is well documented that RLKs can bind either rapid alkalinization factor (RALF) peptides or oligosaccharides that further activate calcium channels. Therefore, there is a need to underpin how pathogens induce RLKs-mediated calcium activation via RALF or oligosaccharide-based activation, and these need further investigation, which will provide novel insights not only for understanding cell wall-mediated plant immunity regulation but also for improving disease resistance [123].

Reactive oxygen species are important signaling molecules that regulate diverse plant growth and biotic and abiotic stress-adaptive responses [124]. In plants, members of the nicotinamide adenine dinucleotide phosphate (NADPH) oxidase family are responsible for ROS production during PTI. It is well known that one of plants’ early responses towards pathogen attack is transient ROS burst, which plays a key role in regulating diverse plant defense responses [125]. During plant–pathogen interactions, the apoplast is a major route of ROS production. After pathogen sensing by RLKs and RLPs, a series of rapid biochemical response occurs, which includes ROS generation. For example, RLKs like PBL1 and BIK1 are necessary for apoplastic ROS production [126] and cytosolic calcium burst [127] as well as for disease resistance to fungal and bacterial and pathogens [126]. ROS waves play a vital role in local and long-distance signaling during plant–pathogen interactions. Among RBOHs, the main contributor to the generation of ROS during innate immunity is RBOHD [128]. Pathogen pattern-induced cytosolic calcium burst is essential for the activation of RBOHD, as transient calcium burst causes conformational changes in RBOHD’s N-terminal EF-hand motifs upon PAMP sensing, and CPK phosphorylation causes RBOHD to produce ROS [127,129]. ROS can also raise the intracellular calcium concentration and activate CPK5, even though calcium and CPKs function upstream of RBOHD activation in pattern-triggered immunity [129]. Interestingly, this reciprocal control between ROS and calcium most certainly plays a major part in the long-distance, cell-to-cell propagation of ROS and calcium known as ROS waves and calcium waves, which are thought to regulate systemic signaling during biotic and abiotic stressors [130]. Future studies are required to further explore calcium and ROS interplay during plant–pathogen interactions and defense activation and how they are regulated by cell wall receptors and other apoplastic signaling molecules, which will provide novel insights for understanding the complexity of the plant immune system. This will also help in improving disease resistance by identifying key players that modulate calcium/ROS-driven immune responses against diverse pathogens.

## 5. Revisiting the Role of Hormones in Plant Defense Response

Plants use sophisticated phytohormone signaling networks as a universal defensive mechanism against pathogen invasion [38,40]. It is well documented that plants undergo hormonal reprogramming to restrict disease progression, but it also plays a key role for plant survival, such as in the reallocation of resources, regulation of cell death, and modification of plant architecture [131]. In contrast, pathogens can also manipulate hormonal signaling pathways that support pathogen growth and disease development [131,132]. Based on the available literature, hormones such as SA, JA, and ET are recognized as primary plant defense hormones that provide disease resistance against diverse pathogens [105]. Recent studies have also reported the role of other hormones such as ABA, auxin, brassinosteroids (BL), auxins, cytokinins (CK), and gibberellins (GA), which play important roles in modulating plant responses to pathogen attack [38]. Interestingly, the interaction of different hormonal signaling pathways is critical for balancing growth–stress tradeoffs, which is crucial for plant survival and adaption.

SA plays a critical role in plant defense against biotrophic and semibiotrophic pathogens by triggering local and systemic resistance [38,39]. At the onset of a primary infection, SA levels rise in local leaves, which, along with other transportable signals, leads to the formation of SAR [133,134]. The SA receptors NPR1 and NPR3/NPR4 were identified, and they are crucial for SA-mediated systemic and local resistance [134]. Plants utilize two distinct routes to synthesize SA from chorismate: either through isochorismate synthase 1 (ICS1) in the chloroplast or via PAL in the cytoplasm [135]. The resultant gene network from the hormone-signaling pathways encompasses multiple transcription factor families; for example, WRKY proteins play a role in activating pathogenesis-related (PR) genes like *PR1*, while MYB factors are crucial in activating genes specific to flavonol biosynthesis within the phenylpropanoid pathway [38]. Phytoalexin-deficient 4 (PAD4) and enhanced disease susceptibility 1 (EDS1) genes are essential for the activation of SA pathways. PAD4 and EDS1 encode proteins that resemble triacyl-glycerol lipases, which are required for SA production [136]. SA is important for defense effector genes and systemic acquired resistance (SAR), as evidenced by *NahG* transgenic plants that break down SA with bacterial salicylate hydroxylase [137]. Furthermore, the SA–ABA interaction, as observed in the FLS2 receptor implicated in the PAMP response of *P. syringae*, activates SA and ABA responses, assisting in pathogen protection through stomatal closure [138].

In plants, JA provides defense response against necrotrophic fungal pathogens and pests [40]. On other hand, both biotrophic and hemi biotrophic viruses produce effectors that can manipulate the JA pathway, thereby increasing plants disease susceptibility [132]. JA and its derivatives, generally known as jasmonates, exhibit different functions and serve as a vital signal mediator in the defense against necrotrophic pathogens [40]. In terms of plant defense, JA not only activates the expression of PR genes [123] but also regulates the synthesis of secondary metabolites including glucosinolates, terpenoids, flavonoids, and phytoalexins [139,140]. In Arabidopsis, the *MYC2*, *MYC3*, and *MYC4* genes regulate the accumulation of JA in response to plant herbivory [141]. MYC2 positively regulates the expression of LOX2/3/4 after treatment with MeJA, and it also controls the expression of JAV1 and JAM1, which act as major regulators of JA biosynthesis and catabolism, respectively. After activation of JA signaling, defense responses are initiated near the wound site or SAR at the uninjured site far from the site of infection. Long-distance transport of JA occurs via vascular bundles from the place of initial synthesis to other parts of the plant. Recent investigations have demonstrated that the JA signaling pathway leads to the activated of downstream responsive genes such as *PR3*, chitinase, and lipoxygenase *LOXs* [142]. The MYC2 transcriptional activator regulates JA-mediated suppression of isochorismate synthase 1 (ICS1), a key enzyme in the isochorismate (IC) pathway, resulting in the induction of genes involved in salicylic acid (SA) metabolism via transcriptional regulation of SNAC-A transcription factors [143]. In *Arabidopsis*, genome-wide association mapping has revealed the role of genes involved in varied JA responses and hormonal interplay. The genes include nuclear-localized type B response regulators (RRB), also known as type B ARR in Arabidopsis, which function as a transcription factor and regulate the expression of CK-responsive genes [142]. According to recent studies, JA’s volatile components, such as methyl-JA, are essential for the systemic wound signaling pathway. The bioactive form of jasmonoyl-L-isoleucine (JA-Ile) in Arabidopsis has also been observed to accumulate in distal leaves following pathogen infection [144]. Several studies have highlighted the role of JA and its related oxylipin metabolites in long-distance signaling [145]. Choi et al. [145] investigated the interconnectedness of microbe-associated molecular patterns (MAMPs) and damage-associated molecular patterns (DAMPs) with JA and oxylipin signaling. Recent studies have also implied the role of JA and oxylipins in the coordination of different defense signaling pathways, such as that of SA, to optimize a plant’s response to a particular stress [146,147]. *JAZ9* and *NOG1-2* interact via a common binding domain and inhibit the interaction between *JAZ9* and *COI1* [121]. Effector-triggered immunity (ETI) is exemplified by the relationship between *JAZ9* and *NOG1-2*, wherein the effector reinstates stomata during bacterial infections, thereby decreasing the wound response.

**Figure 3:**
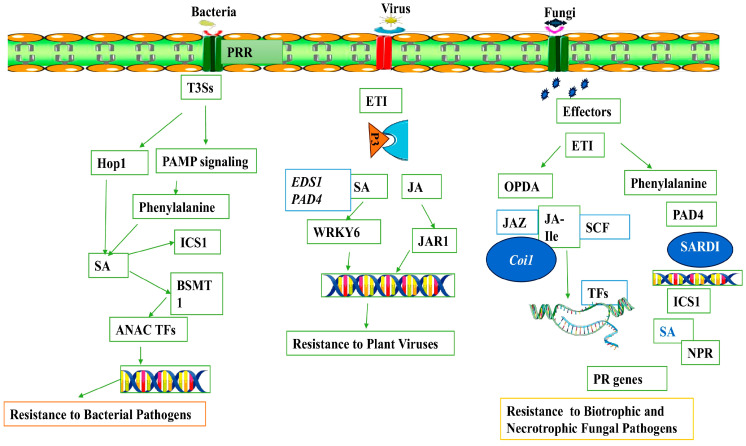
A schematic representation showing SA- and JA-dependent plant immunity against bacterial, viral, and fungal pathogens. This illustration also shows the roles of different players that modulate SA/JA-dependent immune responses.

The role of SA and JA in plant defense against viral pathogens is functionally validated in different plant systems. For example, SA signaling during plant–virus interaction is activated by effector *R* genes that cause the production of reactive oxygen species (ROS) and hypersensitive response (HR) and the expression of pathogenesis-related genes, which confers antiviral disease resistance [148]. After virus infection, the activation of SA-mediated defense response can inhibit intercellular trafficking, replication, and long-distance movement of viral pathogens. The RNA interference (RNAi) pathway is another antiviral defense response associated with the SA-mediated suppression of viral infection [149]. Similarly, the role of JA in plant antiviral defense has been reported in different plant–virus interactions. For instance, Han et al. [150] reported that rice stripe virus (RSV) induces the expression of JA pathway genes, which leads to RSV resistance in rice. Previous study has shown that exogenous treatment of JA decreased the DNA titer of beet curly top virus (BCTV), which further supports the role of JA in antiviral defense [151]. However, contradictory results were also reported that knockout of JA biosynthic genes reduced viral infection and its accumulation [132]. Apart from their respective roles, SA and JA crosstalk plays a crucial role in regulating antiviral defense responses [152]. According to Oka et al. [132], JA biosynthesis enzyme ALLENE OXIDE SYNTHASE (AOS) or JA receptor COI1 silencing boosted plant resistance to TMV and elevated SA levels in COI1- or AOS-silencing plants, which decreased TMV accumulation in tobacco plants. Previous study has also shown the antagonistic interaction between SA and JA in tobacco and *Arabidopsis* plants after viral infection [153]. These findings emphasize the fact that changes in endogenous phytohormone levels are closely correlated with viral movement, replication, symptom development, and defense responses. New insights are being gained into the host manipulation theory and the changes that occur in phytohormones signaling networks during viral infection. Based on the available data, we show how SA and JA provide disease resistance against different types of pathogens in plants in Figure 3.

Ethylene (ET) is a key component of plant immunity in addition to SA and JA. ET primarily confers resistance against necrotrophic fungal pathogens and participates in the induction of systemic resistance mediated by beneficial microbes [154]. Although ET and salicylic acid typically interact antagonistically, plant PRR perception of PAMPs causes ET, SA, and JA to accumulate as well. This trio is necessary for local PAMP-induced resistance to pathogens [155]. Early PTI responses include the production of ET, which regulates the synthesis of downstream defensive proteins and metabolites involved in plant immunity in combination with ROS and the activation of MAPK signaling cascades [156]. The perception of ethylene is initiated at the endoplasmic reticulum membrane. This triggers a signaling cascade that subsequently leads to the transcriptional regulation of ET-responsive genes in the nucleus via the participation of ETHYLENE RESPONSE FACTORs (ERFs) [157]. In response to pathogenic invasion, plants elicit the production of ET, which serves as a key regulator in inhibiting the growth of specific pathogens by modulating the transcriptional activity of genes involved in pathogen response. Plants exposed to a pathogen-associated molecular pattern known as bacterial flagellin peptide 22 (flg22) show the phosphorylation of rate-limiting enzymes involved in ET biosynthesis, ACS2, and ACS6, which is mediated by MAP kinases 3 and 6 (MPK3 and MPK6). Following this, EIN3 triggers the activation of many transcription factors, such as ERF1 and OCTADECANOID-RESPONSIVE ARABIDOPSIS AP2/ERF 59 (ORA59), which are essential in regulating the expression of genes linked to immunity [158]. However, the role of ET in plant immunity is not fully understood. Previous studies have reported that bacterial pathogen *P. syringae* pv. infection in tomato leads to ET production during hypertensive response, which further supports the notion that ET plays a key role in modulating ETI [159]. However, there are major knowledge gaps regarding how ET modulates SA/JA crosstalk and systemic resistance against many pathogens.

Defense hormones have a well-established role in modulating a plant’s response to external stimuli. Plants accumulate a wide range of chemical compounds in response to various stresses, including ABA, which can trigger stomatal closure and increase disease resistance [160]. ABA interacts both antagonistically and synergistically with the ET and SA signaling pathways, respectively, and is implicated in plant responses to a wide variety of diseases [161]. Due to the versatile nature of ABA in mediating plant response to both biotic and abiotic stresses, the role of ABA in mediating plant immunity is well understood. For example, ABA acts synergistically with JA but suppresses SA, which causes plants to be more vulnerable to biotrophic pathogens [162]. Increased levels of ABA in plants facilitate cross-adaptation against plant diseases and drought stress [160]. ABA also mediates the response of JA via the interaction with MYC2 transcription factors [163]. However, ABA also evokes JA responses via interaction with MYC2 transcription factors. JA has a positive interaction with ABA during plant response to multiple stresses and hence activates the MAP kinase signaling pathway in *A. thaliana* [164]. (Similarly, ABA-activated secondary messengers such as reactive oxygen species (ROS), nitric oxide (NO), and cytosolic free Ca^2+^ contribute to plant adaptation to both abiotic and biotic stresses [165]. Hormone crosstalk plays a critical role in regulating the plant immunological network for tailoring immune response to diverse plant pathogens. However, molecular interplay between hormonal cross talk dynamics is not fully understood and therefore warrants future investigation.

## 6. Conclusions

Application of pesticides has been a major driver to control microbial disease, but it has detrimental impact on ecology and human health in addition to the emergence of newly resistant pathogens. Pesticides can also alter soil physiochemical properties as well as soil-beneficial microbiota, which can have a negative impact on plant growth and stress adaptation. Hence, it is important to develop long-term crop disease-resistance cultivars in order to increase crop productivity for the growing population. In this regard, understanding the molecular dynamics of plant–pathogen interactions and identifying potential candidates are key for developing future disease-resistant crops. To increase plant resilience to microbial diseases, scientists are modifying plants’ genetic makeup instead of using chemicals. Incredible discoveries have been made over the past few decades regarding how plants respond to pathogen attack, and a number of important players, including RLKs, calcium channels, RBOHs, and hormonal signatures, have been discovered. However, the details of their fundamental role in plant immunity and their biochemical complexity during plant–pathogen interactions remains largely unknown. Also, how climate change affects plant–pathogen interactions and plant immunity remains enigmatic and warrants future investigation. Because of their rapid natural adaptability to environmental extremes, shorter life cycles, and faster rates of multiplication, phytopathogens may become more common and lead to more severe diseases as a result of climate change. This could result in more catastrophic injury to crop plants. Therefore, understanding how plant immune systems will be affected by climate change and how it affects pathogen distribution and disease severity will help in developing climate- and disease-resistant crops in sustainable agriculture. In the near future, broad-spectrum resistance against pathogens infections is anticipated to be mostly produced by developments in targeted gene insertion by genome editing and molecular stacking. In the future, genome editing, more specifically, CRISPR-based technologies, will play a significant role in enhancing crop resistance to a wide range of pathogens, ensuring food safety and sustainable agriculture.

## Figures and Tables

**Table 1:** List of PRRs identified for pathogen or DAMPs/MAMPs perception in different plants.

Receptors	Family	Co-Receptor/Ligand	Host Plant	References
FLS2	LRR RLK/LLG1	Flg22	*A. thaliana*	[49,50]
EFR	LRR RLK	Elf18	*A. thaliana*	[51]
CERK1	LysM RLK	Chitin	*A. thaliana*, *Oryza sativa*	[52,53]
CEBiP	LysM RLP	Chitin	*O. sativa*	[54]
LYM1/LYM3	LysM RLP	PGNs	*A. thaliana*	[55]
LYP4/6	LysM RLP	PGNs/chitin	*O. sativa*	[56]
LeEix2	LRR RLP	Eix	*Solanum lycopersicum*	[57]
ReMax	LRR RLP	eMax	*A. thaliana*	[58]
PEPR1/2	LRR RLK	Peps	*A. thaliana*	[59,60,61]
Ve1	LRR RLP	Ave1	*S. lycopersicum*	[62]
Cf-2/4/5/9	LRR RLP	Avr2, Avr4, Avr9	*S. lycopersicum*	[63,64,65,66]
Cf-4E	LRR RLP	Avr4E	*S. lycopersicum*	[67,68]
Cf-9B	LRR RLP	Unknown	*S. lycopersicum*	[69]
PSKR1	LRR RLK	PSKα	*A. thaliana*	[70]
BIR1, SOBIR1, ERECTA, SRF3	LRR RLK	Unknown	*A. thaliana*	[71,72]
ds1	LRR RLK	Unknown	*Sorghum bicolor*	[73]
SISERK1	LRR RLK	Unknown	*S. lycopersicum*	[74]
NbSERK1	LRR RLK	Unknown	*Nicotiana benthamiana*	[75]
LYK4	LysM RLK	Unknown	*A. thaliana*	[76]
Bti9, SlLyk13	LysM RLK	Unknown	*S. lycopersicum*	[77]
THE1m, FER	CrRLK1L RLK	Unknown	*A. thaliana*	[78,79]
Pi-d2	LecRK	Unknown	*O. sativa*	[80]
OsWAK1	WAK	Unknown	*O. sativa*	[81]
TaRLK-R1, 2, 3	Other	Unknown	*Triticum aestivum*	[82]
SNC4	Other	Unknown	*A. thaliana*	[83]
LRK10	S-domain	Unknown	*T. aestivum*	[84]
BAK1	LRR RLK	Flg22, elf18, Peps, Eix	*A. thaliana*	[85,86,87,88]
LeEix1	LRR RLP	Eix	*S. lycopersicum*	[89]
SOBIR1	LRR RLK	Avr4, Ve1	*S. lycopersicum*	[90]

**Table 2:** Roles of different types of calcium channels in plant immunity.

Calcium Channel	Family	Activation	Plants	References
CNGC2/4	CNGC family	Flg22, plant elicitor peptide pep3, or lipopolysaccharides (LPSs)	*A. thaliana*	[115,116,117]
OsCNGC9	CNGC family	Chitin	*O. sativa*	[118]
CNGC19	CNGC family	Pep1	*A. thaliana*	[119]
CNGC20	CNGC family	BAK-TO LIFE 2 (BTL2)	*A. thaliana*	[120]
OSCA1.3 and OSCA1.7	OSCA family	BIK1	*A. thaliana*	[114]
ANNEXIN1 (ANN1)	Annexin gene family	CERK1	*A. thaliana*	[121]
GLR2.7, GLR2.8, and GLR2.9	GLR family	Flg22-, elf18-, and pep1	*A. thaliana*	[122]

## Data Availability

Not applicable.
